# Human Epidermal Zinc Concentrations after Topical Application of ZnO Nanoparticles in Sunscreens

**DOI:** 10.3390/ijms222212372

**Published:** 2021-11-16

**Authors:** Zahra Khabir, Amy M. Holmes, Yi-Jen Lai, Liuen Liang, Anand Deva, Michael A. Polikarpov, Michael S. Roberts, Andrei V. Zvyagin

**Affiliations:** 1 Department of Physics and Astronomy & Earth and Planetary Sciences & Clinical Medicine, Macquarie University, Sydney 2109, Australia; zahra.khabir@mq.edu.au (Z.K.); yi-jen.lai@mq.edu.au (Y.-J.L.); liuen.liang@mq.edu.au (L.L.); anand.deva@mq.edu.au (A.D.); 2 ARC Centre of Excellence for Nanoscale BioPhotonics, Sydney 2109, Australia; 3 Clinical Health Sciences and Basil Hetzel Institute for Translational Health Research, University of South Australia, Adelaide 5000, Australia; amy.holmes@unisa.edu.au; 4 National Research Centre “Kurchatov Institute”, Moscow 123098, Russia; polikarpov.imp@gmail.com; 5 Diamantina Institute, University of Queensland, Brisbane 4072, Australia; 6 Centre of Biomedical Engineering, Sechenov University, Moscow 119991, Russia

**Keywords:** nanotoxicology, ZnO nanoparticles, isotope tracing, epidermal zinc, multiphoton microscopy

## Abstract

Zinc oxide nanoparticle (ZnO NP)-based sunscreens are generally considered safe because the ZnO NPs do not penetrate through the outermost layer of the skin, the stratum corneum (SC). However, cytotoxicity of zinc ions in the viable epidermis (VE) after dissolution from ZnO NP and penetration into the VE is ill-defined. We therefore quantified the relative concentrations of endogenous and exogenous Zn using a rare stable zinc-67 isotope (^67^Zn) ZnO NP sunscreen applied to excised human skin and the cytotoxicity of human keratinocytes (HaCaT) using multiphoton microscopy, zinc-selective fluorescent sensing, and a laser-ablation inductively coupled plasma–mass spectrometry (LA-ICP-MS) methodology. Multiphoton microscopy with second harmonic generation imaging showed that ^67^ZnO NPs were retained on the surface or within the superficial layers of the SC. Zn fluorescence sensing revealed higher levels of labile and intracellular zinc in both the SC and VE relative to untreated skin, confirming that dissolved zinc species permeated across the SC into the VE as ionic Zn and significantly not as ZnO NPs. Importantly, the LA-ICP-MS estimated exogenous ^67^Zn concentrations in the VE of 1.0 ± 0.3 μg/mL are much lower than that estimated for endogenous VE zinc of 4.3 ± 0.7 μg/mL. Furthermore, their combined total zinc concentrations in the VE are much lower than the exogenous zinc concentration of 21 to 31 μg/mL causing VE cytotoxicity, as defined by the half-maximal inhibitory concentration of exogenous ^67^Zn found in human keratinocytes (HaCaT). This speaks strongly for the safety of ZnO NP sunscreens applied to intact human skin and the associated recent US FDA guidance.

## 1. Introduction

Advances in the rapidly growing field of nanotechnology have generated several new and effective products now being used in cosmetic formulations [1,2,3], in dermatology [4,5], and in nanomedicine [6,7,8]. However, due to the widespread application of nanotechnology products in industry, medicine and daily life, concerns have been raised as to whether they lead to adverse effects to humans and environment [9,10,11], including previously unknown toxicities [12]. This aspect has led to the field of nanotoxicology, defined as a toxicological assessment of engineered nanomaterials [13].

Zinc oxide (ZnO) nanoparticles (NPs) are widely used in cosmetic products, with 70% of all ZnO NPs now being used in personal care products, including as sunscreens to filter the harmful ultraviolet sunlight radiation (UVA (320–400 nm) and UVB (290–320 nm)) [14]. NPs of ZnO (<30 nm) are typically incorporated into sunscreen formulations as they are aesthetically appealing, appearing transparent on the skin, and are effective in reflecting UV absorption [15]. Typically, ZnO NPs with a diameter between 20 and 100 nm are incorporated into sunscreen formulations [16], which is the size range selected for the stable isotope engineered ZnO NPs designed and used in this study.

Controversy has existed over the last decade regarding the safety of ZnO NPs [17], with concerns raised regarding in vitro studies used to study ZnO NP cytotoxicity [18]. ZnO NPs are suggested to induce cyto- and genotoxicity in human epidermal keratinocytes through DNA damage, intracellular reactive oxygen species (ROS), and oxidative stress [19,20]. However, the cytotoxic concentrations of ZnO NPs vary with the type of immortalised keratinised monolayers and cell culture media used and range from 0.8 to 5 μg/mL in the human epidermal cell line (A431) [21] to 30–50 μg/mL for the HaCaT cell line in DMEM [22]. Typically, ZnCl_2_ or ZnSO_4_ have been used to model soluble Zn salts at equal stoichiometric Zn^2+^ concentrations as a positive control [23,24]. Unfortunately, these studies do not translate to in vivo human skin toxicity as the cytotoxic concentrations are less than or similar to the reported endogenous human epidermal zinc concentrations of 60 µg/g of dry weight [25,26]. Thus, it is apparent that in vitro keratinocyte monolayers may lead to misleading conclusions not relevant to in-use scenarios and the use of in vivo or ex vivo human skin is required to define the realistic safety of ZnO NPs and their dissolved zinc species.

To date, research related to ZnO NPs applied to human skin has been focussed on the extent to which ZnO NPs penetrate human skin after topical application. Gamer et al. [27] and Cross et al. [28] analysed the penetration of ZnO NPs across full-thickness porcine skin and human epidermal membranes, respectively. Zvyagin et al. [29] and Roberts et al. [30] were the first to use non-invasive multiphoton imaging to show that zinc oxide does not penetrate the outermost layer of the skin, the stratum corneum (SC), a finding also reported in later studies [31,32,33,34,35]. According to a published report by the Therapeutic Goods Administration (TGA) of Australia concerning the safety of inorganic sunscreens, ZnO NPs do not cause adverse health effects unless they reach the viable epidermis (VE) [36]. Since their penetration is limited to the SC, systemic absorption and consequent toxicity is unlikely [16,20,28].

The dissolution of ZnO NPs at the sunscreen–skin interface and subsequent permeation of solubilised zinc species throughout the VE has been observed [31]. It has been known for several decades that the human skin has an acid mantle with a pH 4–6 and a pH gradient across the SC ranging from pH 4.5–5.3 at the surface to pH 6.8 in the stratum basale [37]. This can elicit the dissolution of topically applied ZnO NPs and concomitant percutaneous absorption of zinc. Previously, an increase of zinc concentration in human skin has been reported following the exposure to topical formulations containing ZnO [38,39], although conversely, in pig skin after application of microfine ZnO, one study suggested that the levels found were similar to those dosed with the control vehicle [27]. Our work using in vitro human epidermal cells, viable and non-viable ex vivo human skin showed that solubilised zinc species that underwent dissolution can penetrate human skin after topical application of ZnO NPs used in commercial sunscreens even after simulated in use scenario [31,40,41]. To the best of our knowledge, our work showing an increase in epidermal zinc concentrations after application of ZnO NPs are the only in vivo human studies to date [42,43,44].

As zinc is the second-most abundant trace element in the human body [45], Gulson et al. [46] used the traceable isotope ^68^Zn to quantify zinc by inductively coupled-plasma–mass spectrometry (ICP-MS) in blood and urine samples of volunteers exposed to sunscreens containing ZnO NPs in an outdoor setting. They also measured zinc absorption in indoor studies using photochemical-based formulations containing ZnO NPs (mean diameter, ~30 nm; ^68^Zn enrichment, 52%) [47]. They found a small increase of ^68^Zn within volunteers’ blood and urine after a 5-day application, with the highest levels being observed for women dosed with ZnO NPs. As ICP-MS only measures Zn ions after acid digestion of samples, Gulson et al. were unable to determine whether intact ZnO or solubilised zinc species penetrated the skin and the localised skin concentrations were not determined.

**Figure 1:**
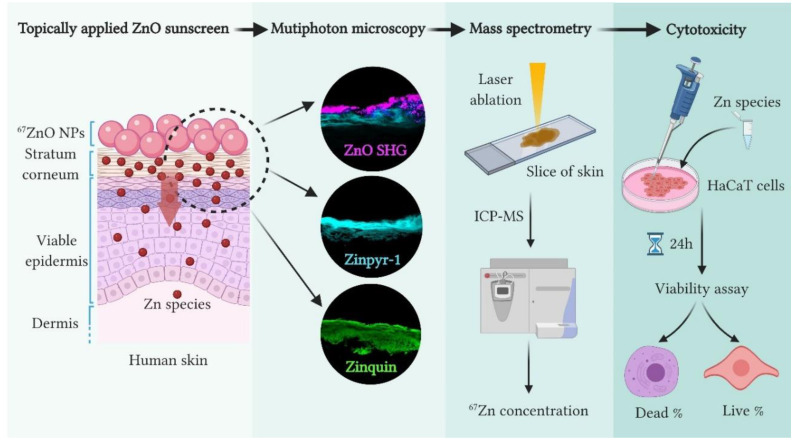
Schematic illustration of techniques used in this study to image and quantify the permeated zinc species formed by ^67^ZnO dissolution on the skin surface. Zinc species were detected by multiphoton microscopy using the second harmonic generation signal of ZnO nanocrystals, by multiphoton and confocal fluorescence microscopies using fluorescent zinc sensors, Zinquin ethyl ester and Zinpyr-1; and mass spectrometry. Created with BioRender.com.

In this work, we applied Gulson’s approach [48] to definitively quantify zinc ion absorption from ZnO NPs into the VE to determine the resulting exogenous zinc concentrations achieved after topical application to intact human skin, as well as to relate them to the endogenous zinc concentrations and to define the exogenous zinc concentrations associated with zinc-induced human keratinocyte (HaCaT cell line) cytotoxicity. We synthesised ZnO NPs enriched with ^67^Zn and investigated the distribution of ^67^ZnO NPs and dissolution products in either excised human skin epidermis (HSE) or freshly excised full-thickness human skin topically treated with ^67^ZnO NP sunscreen formulation. The distribution of ^67^ZnO nanocrystals on the superficial layers of HSE was imaged by using two-photon second harmonic generation (SHG) microscopy characterised by the reduced background [29,49,50]. The distribution of free ^67^Zn^2+^ and ^67^Zn labile species in SC and VE of stained HSE with zinquin and Zinpyr-1 (ZP1), respectively, were acquired by confocal microscopy. ^67^Zn amounts in freshly excised full-thickness human skin were quantified and mapped by ICP-MS and LA-ICP-MS, respectively. Percutaneously absorbed ^67^Zn in VE was compared with IC50 assayed in human keratinocytes (HaCaT cells). Figure 1 illustrates the multi-modal techniques used in this study.

This is the first time, to the best of our knowledge, that the potential human epidermal cytotoxicity of sunscreens that incorporate ZnO NPs has been evaluated based on the exogenous zinc ion concentrations detected in the epidermis. Our results support the view that sunscreens containing ZnO NPs are safe after topical application to intact human skin.

## 2. Results and Discussion

### 2.1. Characterisation of ^67^ZnO-PEG NPs

**Figure 2:**
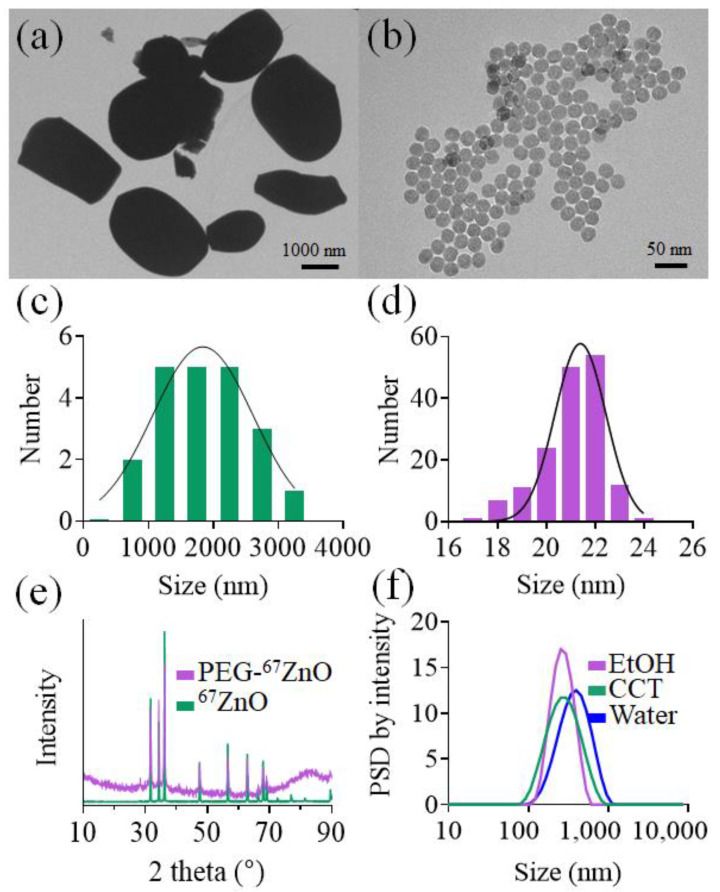
(**a**) TEM image of as-received micron-sized ^67^ZnO powder. Scale bar, 2 μm. (**b**) TEM image of ^67^ZnO-PEG NPs synthesised by the co-precipitation method. Scale bar, 50 nm. Particle size distribution histograms of (**c**) micron-sized ^67^ZnO and (**d**) ^67^ZnO-PEG NPs powders. Solid curves show Gaussian fitting. (**e**) XRD patterns of micro-^67^ZnO and ^67^ZnO-PEG NPs powders. (**f**) Particle size distribution by intensity of colloidal dispersion of ^67^ZnO-PEG NPs in CCT, EtOH and water acquired by dynamic light scattering.

Transmission electron microscopy (TEM) images of micrometre-scale and nanometre-scale ^67^ZnO powders are presented in Figure 2a,b. The as-synthesised ^67^ZnO-PEG NPs were spheroidal in shape with a mean size of 20 nm. The particle size distribution was analysed using TEM images by fitting the histogram with a normal distribution function (Figure 2c,d), yielding FWHM (full width at half maximum), of 4 nm. By controlling the synthesis parameters, as-synthesised NPs were in the diameter range of 20–100 nm (see Supplementary Materials, Section S1, Figures S1 and S2 and Table S1). The acquired and analysed XRD spectra of the micrometre-scale and nanometre-scale ^67^ZnO powders (Figure 2e) showed hexagonal wurtzite crystal structure for both samples, reference code 01-079-207 of the International Centre for Diffraction Data (ICDD). ^67^ZnO-PEG NP XRD peaks were broadened in comparison with micrometre-scale ^67^ZnO. These results show the potential of the reported co-precipitation protocol to produce near monodispersed ZnO NPs with a tuneable size range.

The mean hydrodynamic diameters and (polydispersity indices) of ^67^ZnO-PEG NP suspensions in MQ water, ethanol (EtOH), and capric caprylic triglyceride (CCT) determined by the dynamic light scattering were 341 nm (0.162), 254 nm (0.180), and 248 nm (0.063), respectively (Figure 2f). The difference between the primary size determined by TEM and these results indicated that the NPs aggregated in the solvents: less in CCT, more so in water. Aggregation of ZnO NPs in ultra-pure MQ water is due to low ionic strength of the solvent and a lack of stabilising organic matter [51], whereas in CCT, there is reduced aggregation potentially due to its increased viscosity of the solvent conceivably inhibiting diffusion and particle collisions [52]. However, in aqueous solvent, the dissolution of zinc ions from bulk ZnO (diameter ~1920 nm) was reduced compared to that of ZnO NPs (20–75 nm) due to the increased surface area for dissolution available for the NPs [53]. Therefore, increasing the size of the particles through aggregation would likely decrease the dissolution of ionic zinc species [54], this effect would be reduced for CCT due to lower observed aggregation and that it is a non-aqueous solvent.

### 2.2. Multiphoton Microscopy Imaging

**Figure 3:**
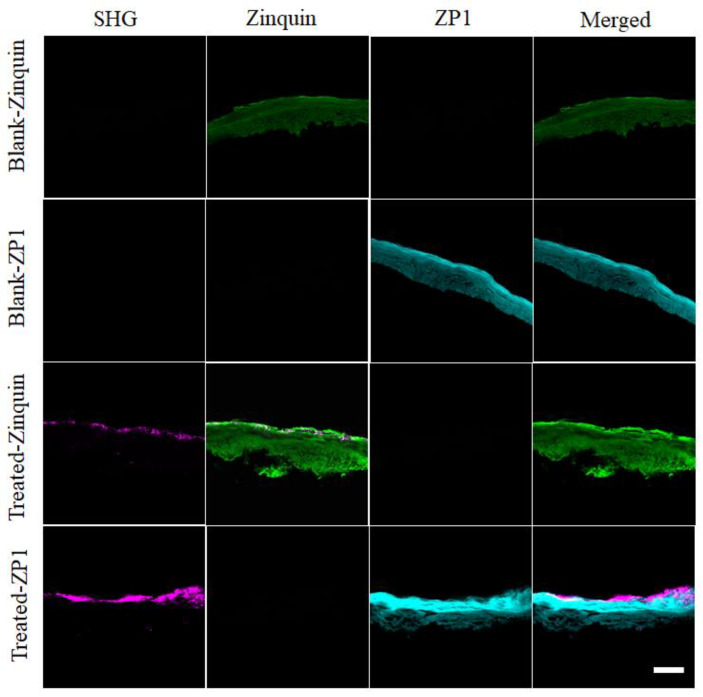
Multiphoton and confocal microscopy images of cross-sectioned HSEs post-treatment with either blank CCT formulation (Blank) or ^67^ZnO-PEG (20 %*wt*/*wt*) in CCT formulation (Treated) for 48 h at 35 ± 1 °C. First and second rows show the images of blank HSEs stained with zinquin and ZP1, respectively. Third and fourth rows are the images of treated HSEs stained with zinquin and ZP1, respectively. From left, first, second, third and fourth columns show images of HSEs in SHG (magenta), zinquin (green), ZP1 (cyan) and merged channels, respectively. SHG signal of nano-sized ^67^ZnO-PEG was acquired with multiphoton excitation/emission at 800 nm/400 nm. Zinquin signal was obtained with multiphoton excitation/emission at 740 nm/490 nm. For ZP1, excitation/emission was collected at 488 nm/540–560 nm using a 488-nm argon laser. Scale bar, 40 mm.

**Figure 4:**
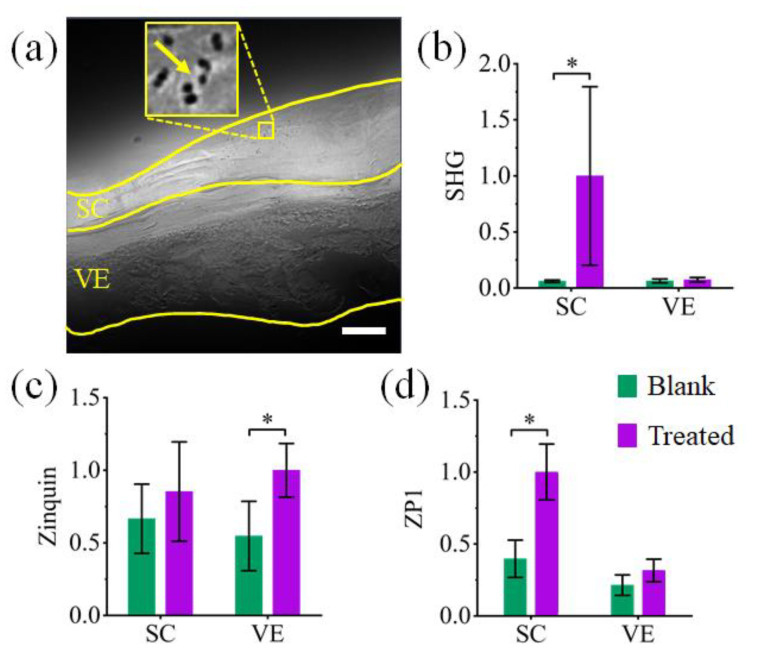
(**a**) Structured illumination microscopy image of ZnO-treated HSE. The inset is a magnified area of the image showing ^67^ZnO-PEG NPs aggregates (pointed by a yellow arrow) on SC surface. Scale bar, 5 μm. Results of image analysis of SHG, zinquin and ZP1 signals in untreated (Blank) and ^67^ZnO-treated-HSEs (Treated). Bar plots compare the normalised intensities (mean ± 95% confidence interval, *n* = 7) of (**b**) SHG, (**c**) zinquin, and (**d**) ZP1 signals in SC and VE of blank and treated HSEs. (*) shows significance difference levels at *p* < 0.05.

Multiphoton and confocal microscopy images of HSE are shown in Figure 3. SHG images (magenta) are shown in Figure 3, as observed in HSE treated with the ^67^ZnO-PEG NPs in CCT formulation (hereafter, “treated”). At the same time, no SHG signal was detected in the fluorescent channels spectrally configured for ZP1 (cyan) and zinquin (green). SHG signals were confined within the outer layers of the SC of the treated HSE, and not in the VE. Considering SHG originated exclusively from ZnO nanocrystals, this result indicated that no NPs penetrated the SC into VE. HSE (dermis removed) was used in these experiments to remove the inherent SHG signal of the collagen found throughout the dermis of human skin. Confocal microscopy images of the blank and treated HSE stained with zinquin and ZP1 zinc sensors are shown in Figure 3 and reported on the presence of intracellular and labile zinc species in HSE, respectively. It is important to note the positive fluorescence contrast of both zinquin- and ZP1-stained HSEs in both SC and VE. The contrast increased in the treated HSE in comparison with the blank HSE, as shown in Figure 3. Superimposed images of SHG and zinc sensors clearly delineate the distribution of ^67^ZnO-PEG NPs in SC and ZnO dissolution species across the treated HSE. The results of the multiphoton microscopy were also confirmed by structured illumination microscopy, as shown in Figure 4a. ^67^ZnO-PEG aggregates were detected across the superficial layers of the SC (spots marked by a yellow arrow) and no penetration was observable beyond the SC.

To evaluate the uptake of zinc species in the treated HSE, we plotted the fluorescence intensities of the zinc sensors and SHG intensity averaged over the SC and VE layers of the treated and blank HSE in Figure 4b–d. The SHG signal was found to be negligible in the VE of both treated and blank HSE, although as expected it was significantly greater in the SC of the treated HSE in comparison with the negligible SHG in the blank HSE’s SC (Figure 4b). The zinquin and ZP1 fluorescent signals were enhanced in the treated HSE in comparison with that of the blank HSE (Figure 4c,d). A statistically significant increase of the ZP1 fluorescence (*p* ≤ 0.05, *n* = 7) in the SC of the treated HSE indicated an enhanced uptake of labile zinc species within the SC, following the dissolution of ^67^ZnO-PEG NPs. This observation is supported by the observed zinc levels in the treated SC using X-ray fluorescence microscopy technique [55]. It has been reported that zinc sequestration into zinc-containing proteins diminishes the ZP1 fluorescence [56]. The reduced ZP1 fluorescence in the VE of the treated and blank HSE suggests that labile Zn was sequestered by intracellular proteins in VE as shown by no increase in ZinPyr-1 intensity in Figure 3. This inference was corroborated by the increased signal in zinquin fluorescence resulting from the increase in bound intracellular zinc in the treated HSE VE. Zinquin is known to be less discriminate between free, labile and bound intracellular zinc species as it measures all intracellular zinc [57], and as such, revealed a statistically significant uptake of exogenous zinc in VE resulting from ^67^ZnO-PEG NP topical application on excised skin.

We note that, in general, fluorescence assays of zinc sensors provide quantitative evaluation of the zinc concentration relative to a control which can be used for comparison between treated and blank skin samples. To assess the potential cytotoxic effects of Zn^2+^ and zinc species, quantitative measurements of the zinc uptake in treated and blank skin were performed [58].

### 2.3. Quantitative Measurement of Absorbed ^67^Zn in Skin Layers by LA-ICP-MS and ICP-MS

**Figure 5:**
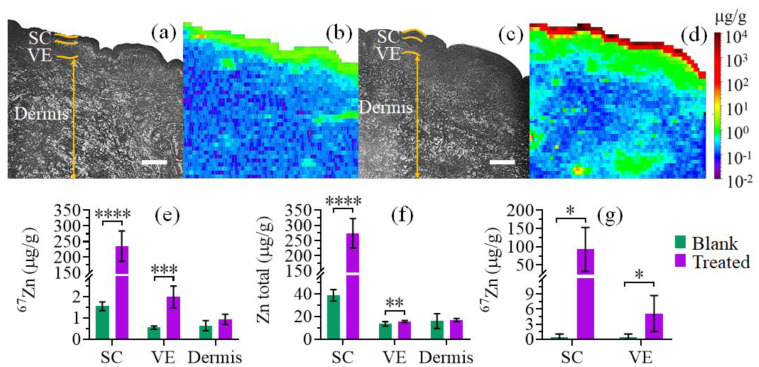
Results of mass spectrometry analysis of untreated and ^67^ZnO-PEG NPs treated human skin samples. (**a**) Brightfield image of full-thickness freshly excised human skin section post-treatment with blank CCT formulation (Blank) for 48 h at 35 ± 1 °C. SC, VE and Dermis designate stratum corneum, viable epidermis and dermis layers, respectively. (**b**) LA-ICP-MS map of ^67^Zn concentration (μg/g) of (**a**). (**c**) Brightfield image of full-thickness freshly excised human skin section post-treatment with ^67^ZnO-PEG NPs (20 %*wt*/*wt*) in CCT formulation (Treated) for 48 h at 35 ± 1 °C. (**d**) LA-ICP-MS map of ^67^Zn concentration (μg/g) of (**c**). (**e**) ^67^Zn and (**f**) total zinc concentrations in skin measured by LA-ICP-MS for blank and treated samples. (**g**) ^67^Zn concentration measured by solution-based ICP-MS for untreated and treated HSEs. (*), (**), (***) and (****) show significance difference levels at *p* < 0.05, *p* < 0.01, *p* < 0.001 and *p* < 0.0001, respectively. Data are presented as mean ± 95% confidence interval, n^3^ 4. Scale bars are 200 μm.

LA-ICP-MS analysis was carried out using cross-sectioned freshly excised full-thickness human skin treated with either blank CCT (blank skin) or ^67^ZnO-PEG NPs (20 %*wt*/*wt*) in CCT (treated skin). Figure 5a,c shows the brightfield images of cross-sectioned blank and treated skin samples, respectively. The results of LA-ICP-MS analysis are presented as an elemental map of ^67^Zn for blank (Figure 5b) and treated skin (Figure 5d). Figure 5e presents the mean concentrations of ^67^Zn tracer in ^67^ZnO-PEG treated and blank skin sections determined by LA-ICP-MS. A considerable amount of ^67^Zn was found in the treated skin SC. ^67^Zn concentration, CZ 67n gradually reduced from the uppermost layers downwards: 235 ± 49 μg/g of dry tissue in SC to 2.0 ± 0.5 μg/g in VE in the treated skin. ^67^Zn concentration in dermis of the treated skin was measured as 0.9 ± 0.3 μg/g and deemed indistinguishable from that of the blank skin dermis (0.6 ± 0.2 μg/g). The concentration of exogenous zinc, $C_{exZn}$ in skin layers was calculated using CexZn=[CZ 67n (treated)−CZ 67n (control)]/E67, where $E_{67}$—the enrichment factor of ^67^Zn tracer determined in a separate ICP-MS measurement as 65.4 ± 0.2%, with the results summarised in Table 1.

The endogenous concentration of ^67^Zn tracer in blank skin VE was found to be CZ 67n (control)= 0.5 ± 0.1 μg/g from where the concentration of endogenous total zinc, $C_{enZn}\;$ was estimated using CenZn=CZ 67n (control) /A67, where $A_{67}$ represents the natural abundance of ^67^Zn isotope tabulated as 4.04% [59]. The total zinc concentrations in SC, VE and dermis of blank skin were estimated as 39 ± 5 μg/g, 13 ± 2 μg/g, and 16 ± 7 μg/g respectively. This represents an approximately ten-fold increase in the elemental zinc amount accumulated in SC of treated skin (CZ 67n (treated) = 235 ± 49 μg/g) because of the ZnO NP sunscreen application, with the exogenous zinc combined as ZnO NP aggregates, Zn^2+^ and zinc species. In VE of the treated skin, we detected a statistically significant increase of the total zinc concentration by ca. 23% (Figure 5f), the exogenous zinc fraction comprised Zn^2+^ probably existed in protein-bound forms. The exogenous zinc accumulation in dermis was statistically insignificant.

The measured concentration of endogenous epidermal zinc 13 ± 2 μg/g does fall in the range of the reported values [25], but these values vary widely, ranging from 3 to 60 μg/g depending on the analysis technique [26,60]. To validate our results reported here, we carried out a separate experiment to assay $C_{enZn}$, using homogenised untreated human skin by using “gold standard” quantitative technique ICP-MS and its modality LA-ICP-MS (see Section S2, Supplementary Materials). $C_{enZn}$, measured by ICP-MS was 15 ± 4 μg/g. $C_{enZn}$ measured by LA-ICP-MS averaged over several sites of the homogenised human skin sample was 11 ± 8 μg/g. $C_{enZn}$ measured by ICP-MS and LA-ICP-MS were consistent with each other and consistent with the value of 13 ± 2 μg/g reported here underpinning the reliability of our results.

To validate the results obtained by LA-ICP-MS of cross-sectioned freshly excised skin, we carried out solution-based ICP-MS of tape-stripped HSEs, with the results presented in Figure 5g. Most of the ^67^Zn tracer was recovered within the wash (630 ± 110 μg/g) and is therefore considered unabsorbed. In comparison with the blank HSE, a significant increase of ^67^Zn in the treated SC was detected in good agreement with the data by the LA-ICP-MS analysis and fluorescence zinc assays (c.f. Figure 3). ^67^Zn recovered from the remaining VE after tape-stripping was ca. 4000-fold greater in the treated HSE compared to the blank, amounted to CZ 67n = 4.5 ± 4.0 μg/g. This value was almost twice of CZ 67n in VE measured by LA-ICP-MS. This discrepancy was attributed to incomplete removal of the SC containing ZnO NPs from the HSE due to an insufficient number of tape-strips—we applied 3 tape-strips due to the HSE frailty in comparison with the required number of 20 or even 40 tape-strips [61]. Therefore, $C_{exZn}$ in VE of the treated HSE derived from the ICP-MS data was overestimated. Ex vivo human skin used in the ICP-MS study was not viable, and therefore the skin’s innate zinc homeostatic system was impaired, including active transport of an excess of zinc to peripheral microcirculation. The amount of ^67^Zn found in the receptor fluid of the treated samples was comparable with that of the background and indicated that no significant amount of topically applied ^67^Zn permeated across the HSE to the receptor fluid during the 48-h observation period.

Our results offer an insight into how zinc appeared in the systemic circulation, following ZnO NP sunscreen topical application on volunteer’s human skin [46]. The elevated ^67^Zn levels could be found within the furrows and superficial layers of the SC, which were attributed to undissolved ^67^ZnO-PEG NPs. At the same time, the SC of skin contains a significant amount of keratin characterised by abundant sulfhydryl groups of proteins [62]. The high binding propensity of these groups to labile zinc could contribute to the significant Zn concentration detected in the SC. We speculate that the high amount of Zn in the SC acted as a depot for the epidermal permeation of zinc. Previously, this depot led to a long lag of 5 days for the systemic absorption of zinc in human volunteers [46]. Our LA-ICP-MS, ICP-MS and multiphoton microscopy data were consistent and indicative of the presence of the elevated exogenous Zn levels in the VE layers stemmed from Zn species desorbed from ZnO NP-based sunscreen.

### 2.4. Cytotoxicity of ^67^ZnO-PEG NPs in HaCaT Cells

**Figure 6:**
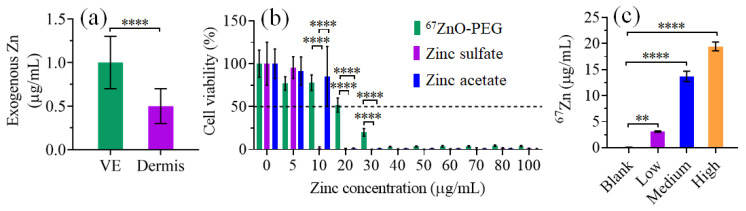
(**a**) Exogenous CZ 67n  expressed in μg/mL, as assayed by LA-ICP-MS in VE and dermis layers (replotted from Figure 5f). (**b**) HaCaT cell viability exposed to zinc acetate, zinc sulphate, and ^67^ZnO-PEG NPs for 24 h. The data format: mean ± SD for *n* = 8 replicates. (**c**) CZ 67n  from supernatant of HaCaT cell-mediated culture media on addition of ^67^ZnO-PEG NPs measured by ICP-MS. Blank, Low, Medium and High stand for cell incubation with 0, 5, 25 and 50 μg/mL of ^67^ZnO-PEG NPs, respectively. The data format: mean ± SD, *n* = 3. (**) and (****) show significance difference levels at *p* < 0.01 and *p* < 0.0001, respectively.

Zinc has specific roles in skin functions and can be detrimental or toxic if present in excessive amounts [63]. The detected Zn species raised a question of whether the excess of zinc that permeates the skin is cytotoxic to keratinocytes within the VE or not? To determine ZnO NP cytotoxicity to keratinocytes, we performed cell viability assays versus Zn concentration. To compare IC_50_ value expressed in terms of μg/mL with the values in terms of μg of ^67^Zn per gram of dry tissue (μg/g), μg/g units were converted to μg/mL by using the reported value of the water content in VE (ca. 70%) (see Section S3, Supplementary Materials) [64]. Figure 6a shows data taken from Figure 5f and replotted versus μg/mL, while the exogenous ^67^Zn concentrations in VE and dermis were recalculated as 1.0 ± 0.3 μg/mL and 0.5 ± 0.2 μg/mL, respectively.

Figure 6b presents the results of the MTT assay of keratinocyte cells (HaCaTs) treated with ionic Zn sources, zinc acetate, zinc sulphate, as well as ^67^ZnO-PEG NPs, in the concentration range of 0–100 μg/mL. The applied concentrations were expressed in terms of the equivalent concentrations of Zn. Zn salts and ^67^ZnO-PEG NPs were not cytotoxic at a concentration of <5 μg Zn/mL, although the cell viability was slightly less for ^67^ZnO-PEG NPs at this concentration. The cell viability dropped at 5–10 μg Zn/mL and 10–20 μg Zn/mL for zinc sulphate and zinc acetate, respectively. The greater cytotoxicity of zinc sulphate observed here was corroborated by in vitro studies using PC12 cells and explained by the greater cellular uptake of zinc [65]. The cytotoxicity of ^67^ZnO-PEG NPs was characterised by IC_50_ = 26 ± 5 μg Zn/mL (equivalent to 25 μg/mL of ^67^ZnO NPs), and appeared less in comparison with that of Zn salts in agreement with literature [66,67].

IC50 of ^67^ZnO-PEG NPs assayed in complete cell culture medium is not directly comparable with $C_{exZn}$ assayed in VE by LA-ICP-MS, because foetal bovine serum (FBS) supplement in cell cultures and extracellular matrix in skin mediates ^67^ZnO-PEG NP interaction with keratinocytes via two processes. Firstly, ZnO NP solubility depends on the matrix of culture medium [53]. In general, ZnO NP solubility is high in an aqueous solution, yielding mostly Zn^2+^, with almost complete dissolution achieved at <10 μg/mL. Secondly, a fraction of the dissolved Zn^2+^ species is sequestered by serum proteins or ligands thus reducing the concentration of potentially cytotoxic species-free zinc. For example, albumin added to complete culture medium in molar excess to Zn^2+^ markedly reduced labile Zn^2+^ to the nM range [68] and addition of bovine serum albumin or EDTA to culture media can chelate excess labile zinc reducing cytotoxicity of zinc species to HaCaT cells [41]. It has been reported that 20 μg/mL of ZnO NP yielded ~5 μg/mL of free zinc in complete cell culture media (RPMI1640 supplemented with 10% FBS) and equilibrated at >40 μg/mL of ZnO NPs to yield 10 μg/mL of free zinc [24].

We investigated the production of potentially cytotoxic species in complete culture medium (DMEM + 10% FBS) qualitatively and quantitatively by fluorescent zinc sensor (ZP1) and ICP-MS assays, respectively. HaCaT cells in DMEM + 10% FBS were dosed with Blank, Low (5 μg/mL), Medium (25 μg/mL) and High (50 μg/mL) concentrations of ^67^ZnO NPs for 24 h, followed by ultracentrifugation to remove cells and serum proteins. The resultant supernatants containing Zn^2+^ and zinc species bound to low molecular weight ligands (termed here, labile zinc, laZn) were assayed. Qualitative evaluation of $C_{laZn}$ based on the fluorescent intensities of zinc sensor ZP1 in the complete cell culture medium (Figure S3) indicated a monotonic increase of the concentration of labile zinc versus the dosed amount of ^67^ZnO and supported the increased cytotoxicity versus concentration of zinc oxide nanoparticle reported previously [24].

The results of the ICP-MS assay of CZ 67n are presented in Figure 6c. The concentration of $C_{laZn}$ can be calculated by using equation ClaZn=CZ 67n /E67. The low dosing (5 μg/mL of ^67^ZnO-PEG NPs) resulted in a 60-fold increase of $C_{laZn}$ and corresponded to an ~25% viability drop (c.f. Figure 6b). The high dosing (50 μg/mL) yielded $C_{laZn}$ = 31 ± 2 μg/mL (CZ 67n= 20 ± 1 μg/mL) and reduced the cell viability to ~50% level (c.f. Figure 6b). Medium to high dosing range yielded a narrow $C_{laZn}$ range of 21–31 μg/mL (CZ 67n, 14–20 μg/mL)—also seen in Figure S3—but resulted in a dramatic viability drop from ~50% to 2%, as obvious in Figure 6b within the $C_{Zn}$ range of 20–50 μg/mL. This suggests that the Zn threshold for significant cell toxicity within the culture media falls between $C_{laZn}$ range of 21–31 μg/mL per addition of 25–50 μg/mL ^67^ZnO-PEG NP.

### 2.5. Discussion

The main objective of this study was to quantify the amounts of labile zinc within the VE after topical application of ZnO NPs to excised human skin. For the first time, to the best of our knowledge, the concentration of exogenous Zn ions in viable epidermis of human skin was measured using a rare stable ^67^Zn isotope tracing technique and benchmarked against the cytotoxicity threshold to support a notion of ZnO sunscreen safety. We observed that ^67^ZnO-PEG NPs were localised and retained in the superficial layers of the SC and did not penetrate further into the VE. A statistically significant increase in the signal intensity of a labile zinc-specific probe (ZP1) post ^67^ZnO-PEG NP application within the SC and a statistically significant increase in the signal intensity of an intracellular zinc probe (zinquin) within the VE supports our hypothesis that zinc species from ZnO NP permeated across the SC to the VE. While most of the zinc was not absorbed percutaneously, an increase of labile zinc was found within the VE and could therefore cause localised toxicity or become systemically available. However, the cytotoxicity is dependent on the speciation of the zinc, its sequestration by extracellular proteins and cellular uptake. We measured Zn concentrations in the VE and crucially found that it was ca. >25-fold lower than the cytotoxicity threshold for ^67^ZnO-PEG NPs. Furthermore, ZnO NPs did not permeate beyond the SC barrier to the viable keratinocytes, thus evading physical damage caused by the NPs to the cell membranes [69].

The ^67^Zn concentration recovered between the IC50 and IC90 concentrations of ZnO-PEG NPs in vitro was found to be significantly, at least 15 times, higher than the exogenous zinc concentration permeated within the VE. Interestingly, Yamada et al. [18] estimated that the human epidermal Zn concentrations after a maximum FDA-approved ZnO NP dose in a sunscreen product is about 10 to 50 times lower than that causing toxicity in human epidermal cells. While intact human skin shows a good safety profile it may not stand for an impaired skin barrier as previous work has shown that dissolved zinc species can significantly increase within the VE after topical application of ZnO NPs to impaired excised human skin [40]. We did not evaluate the dissolution of uncoated ZnO NPs versus PEG-ZnO NPs specifically. According to the literature, the aqueous solubility of PEG-coated ZnO is comparable to that of uncoated ZnO nanoparticles. However, in cell culture medium (RPMI-1640) supplemented with FBS, the solubility of ZnO is almost twice that of PEG-ZnO nanoparticles [70].

## 3. Material and Methods

### 3.1. Synthesis of ^67^ZnO-PEG NPs via a Co-Precipitation Method

All reagents were of analytical grade and used as received without further purification. Acetic acid (CH_3_CO_2_H, ≥99.7%), poly(ethylene glycol) (PEG; average molecular weight 200 Da), potassium hydroxide (KOH, ≥85%), cyclohexane (99.5%), isopropyl alcohol (≥99.7%), acetone (≥99.5%), were obtained from Sigma-Aldrich Chemicals (Sydney, NSW, Australia). Ethanol (EtOH; 100% undenatured) was supplied from Chem-Supply (Adelaide, SA, Australia). Micron-sized ZnO powder enriched to >65% ^67^Zn and a chemical purity >99.96% was supplied by FSUE Integrated Plant Electrohimpribor (Lesnoy, Russia), and produced by an electromagnetic enrichment method (calutron).

A top-down chemical approach based on the co-precipitation method was used to etch ^67^ZnO micrometre-scale particles to nanoscale ^67^ZnO particles (see Supplementary Materials, Section S1, for details). The precursor concentrations and time were optimised by using commercial ZnO powder. Briefly, as-received ^67^ZnO micro-particles were mixed with acetic acid at the molar ratio of 1:2 heated to 50 °C to produce enriched zinc acetate ^67^Zn(Ac)_2_ salt, which was dried at the same temperature for 2 h. Then, 10 mL of 0.1-M ^67^Zn(Ac)_2_ was mixed with 0.7-mL PEG and stirred for 30 min at room temperature. Then, 5 mL of 1 M KOH solution was added dropwise and stirred at room temperature to obtain white gel, which was transferred to a water bath preheated to 75 °C. The precipitate of formed ^67^ZnO-PEG NPs was heated for 1 h to complete the reaction. The precipitate was collected and washed several times with EtOH.

To produce sunscreen formulation mimicking commercially available sunscreens, 20 %^67^ZnO-PEG (*wt*/*wt*) was mixed with capric caprylic triglyceride (CCT). To this aim, EtOH solution of ^67^ZnO-PEG NPs was centrifuged at 10,000 rpm for 10 min, and re-dispersed in isopropyl alcohol, followed by re-dispersion in acetone, cyclohexane, and finally in CCT.

### 3.2. Characterisation of ^67^ZnO-PEG NPs

The size and morphology of micrometre and nanometre-scale ^67^ZnO materials were determined by TEM (PhilipsCM10, Eindhoven, The Netherlands) operated at the accelerating voltage of 100 kV. To prepare the TEM sample, ^67^ZnO dry powder was dispersed in EtOH in the concentration of 1 mg/mL, dropped on a copper grid and allowed to dry. The crystal structure of as-synthesised ^67^ZnO-PEG NPs was determined by an X-ray diffractometer (PANalytical X’Pert Pro MPD) with a Cu Kα X-ray radiation source at the characteristic wavelength of 1.5418 Å. The data were collected over an angular range of 10–80°, step size 0.017°. The hydrodynamic diameter and polydispersity index of ^67^ZnO-PEG NPs dispersed in CCT, EtOH and MQ water in polystyrene cuvettes (Sigma-Aldrich, Sydney, NSW, Australia) were measured using a dynamic light scattering instrument (DLS; Zetasizer NS, Malvern, UK). ^67^ZnO NPs in CCT, EtOH, and MQ water were prepared at the concentration of 1 mg/mL and sonicated for 30 min.

### 3.3. Preparation of Heat-Separated Human Epidermis Samples

Human skin patches were excised from female donors (*n* = 3, aged 46–53 years) undergoing abdominoplasty surgical procedures. Prior to the research, informed written consent was obtained from all participants (The Queen Elizabeth Hospital, ethics approval protocol-2009208). The subcutaneous fat was removed by blunt dissection, and the skin samples were placed in a water bath at 60 °C for 90 s to separate the epidermis from the dermis. The human skin epidermis (HSE) was then floated on water and placed onto a filter paper to dry. The processed HSE samples were kept in ziplock bags at −20 °C and were used within 6 months.

### 3.4. Skin Penetration Assay

After thawing HSE, if necessary, 3-cm-diameter disks were cut out from the skin patches and mounted on static Franz diffusion cells. The receptor and donor chambers were filled with 3.5 mL and 1 mL of PBS buffer (pH 7.4), respectively, and placed in a water bath at 35 °C for 30 min to equilibrate. The transepithelial electrical resistance was measured (>20 kW) to ensure the HSE sample integrity according to Cross et al. [28]. To measure transepidermal water loss, PBS buffer was removed from the donor chamber and the skin surface was blotted dry. The measurements were carried out using an Aquaflux instrument (Biox Systems, London, UK) at 23.2 °C and 44.5% humidity to select HSE membranes with values <40 g/m^2^/h to ensure the skin barrier integrity. The PBS buffer was removed and replaced with 3.5 mL of HEPES buffer that was used as receptor fluid.

The HSEs were dosed at 2 mg/cm^2^ of either 20 %*wt*/*wt* ^67^ZnO-PEG NPs in CCT or CCT labelled as treated and blank, respectively, and then incubated at 35 ± 1 °C on a submersible magnetic stirrer for 48 h. We covered HSE surface by the formulation entirely avoiding direct contact with the skin sample. The decreased viscosity of the sunscreen formulation at 35 °C facilitated the uniform spreading. 0.5 mL of the receptor solution was sampled at time points 0, 0.5, 2, 4, 8, 24, and 48 h, and the receptor solution was replenished. The unabsorbed ^67^ZnO-PEG formulation was washed away after 48 h using a natural sponge immersed in PBS and then blotted dry.

Dried HSEs were weighed, and a tape stripping procedure was then performed three times to remove most of SC [71]. Tape strips, remaining HSE and wash sponges were weighed and transferred to labelled acid-resistant tubes to be digested for ICP-MS analysis.

### 3.5. Sample Preparation for Microscopy

Human HSE was used in the microscopy experiments to remove the collagen within the dermis that emits the same SHG signal as ZnO NPs. HSEs were cut into 2 × 2 mm^2^ pieces and embedded in optimal cutting temperature medium, which were then snap-frozen at −80 °C, sectioned into 10-µm-thick slices using a cryostat (Leica. CM 1950, Nussloch, Germany) and mounted on poly L-lysine-coated microscope slides. The slide mounted HSE samples were stained with 10 μL of ZP1 or zinquin following a protocol reported elsewhere [31]. An excess of the staining solution was removed after 10 min and rinsed twice with ultrapure MQ water. Finally, the sections were covered with coverslips and sealed for microscopy observations.

### 3.6. Multiphoton Laser-Scanning Microscopy

Stained HSE sections were imaged using a Zeiss LSM710 multiphoton microscope equipped with an argon gas laser (488 nm) and a tuneable titanium-sapphire femtosecond pulsed laser (Mai Tai, Spectra Physics, Stahnsdorf, Germany). A high numerical aperture objective (40×/NA1.1) was used. The SHG signal of dispersed ^67^ZnO-PEG NPs in CCT was detected at 400 nm by using the excitation at 800 nm (6.3 mW). The limit of quantification (LOQ) of ZnO NPs was previously determined by Leite-Silva et al. [33]. ZP1 and zinquin fluorescent sensors were excited by the argon (488 nm (0.7 μW)) and femtosecond (740 nm (6.3 mW)) lasers, with emission detected at 540 nm–560 nm and 490 nm, respectively. To achieve two-fold spatial resolution, super-resolution microscopy was carried out using a structured illumination module Zeiss Elyra PS.1 integrated into the laser-scanning confocal microscope using PlanAPOCHROMAT 100×/1.46 oil-immersion objective. For each condition, 3 samples were imaged. Image analysis was conducted using Image-J and ZEN lite software (Sigma-Aldrich, Sydney, NSW, Australia).

### 3.7. LA-ICP-MS Analysis

Freshly excised abdominal human skins were received from female donors (*n* = 3, aged 27–47 years) who had undergone abdominoplasty surgery. Before the research, informed written consent was obtained from all participants (The Macquarie University Hospital, ethics approval protocol-5201200935). The skin samples were processed for the subcutaneous fat removal and used for permeation assay on the same day. The permeation assay was performed using the same Franz cells set-up and formulations described in Section 3.4, with the incubation time changed to 24 h. Afterwards, untreated (blank) and treated skin samples were snap frozen and cut into 50-μm slices perpendicular to the skin surface, mounted on a glass slide and placed in a clean container to avoid contamination. Ultimate care was taken to cut the samples to avoid smearing the remnant sunscreen across the sample. In situ measurement of ^67^Zn concentration in the skin samples was carried out using a Photon-Machines Analyte Excite 193 nm Excimer laser ablation system with Helex II sample chamber coupled to an Agilent 7700× quadrupole ICP-MS system at Macquarie Geoanalytical (MQGA) Centre, Macquarie University. Helium was used as the carrier gas (flow rate, 0.8 L/min) mixed with argon (flow rate, 0.8 L/min) before introducing it to a torch in ICP-MS. To quantify the LA-ICP-MS measurement, a spiked gelatine sample with a defined concentration of ^67^Zn (10 μg/g) was produced as described elsewhere [72] and used as standards to quantify ^67^Zn concentrations in skin. The ^67^Zn-spiked gelatine samples were collected for acid digestion and their ^67^Zn concentrations were determined accurately and precisely using Solution-nebulisation ICP-MS. A carbon isotope ^13^C was used as the internal standard to correct for differences in the ablation yield of the materials, plasma condition, and instrumental drift. Laser energy of 2.11 J/cm^2^ and a repetition rate of 10 Hz were used to maintain an adequate signal-to-noise ratio of ^13^C. LA-ICP-MS was operated in a line-scan mode by rastering a square spot 35 × 35 μm^2^ at the 35 μm/s scan rate. The data compression was carried out using software Iolite (version 3.6).

### 3.8. Sample Preparation for Solution Nebulisation ICP-MS

The collected receptor fluid samples, natural sponges from the wash, remaining HSEs, tape strips and gelatine standard were prepared for ICP-MS assaying using a standard addition technique. The collected receptor fluids were digested by adding 4.7 mL of 2% (*v*/*v*) HNO_3_ (69% ultra-pure grade for ICP-MS, Choice Analytical, Australia) to 0.3 mL of each receptor fluid to make up solution volume of 5 mL. To digest the tapes and remaining HSE, 6 mL of HNO_3_ (69% ultra-pure grade for ICP-MS, Choice Analytical, Australia) and 1 mL of H_2_O_2_ (Westfarmer Chemicals, 30% *v*/*v*) were added to each. To digest the sponges, 3 mL of HNO_3_ was added. Afterwards, the samples were heated up in a heater block by increasing the temperature gradually from 35 °C to 100 °C until digestion of organic compounds was completed, and bubbling stopped. The collected gelatine samples were, first, mixed with 1 mL of HNO_3_ and heated at 60 °C for 3 h. All acid-digested samples were diluted with ultrapure MQ water to make the acid content ≤2% (*v*/*v*) and aliquoted accordingly. For the calibration curve sets, the aliquots of the acid-digested samples were spiked with a certified ^67^Zn standard solution (IRMM^®^ Certified Reference Material, Sigma-Aldrich, Sydney, NSW, Australia) at different concentrations. A calibration set of the skin assay samples was 0, 10, 20, 50, 100, 200 and 500 ppb; and 0, 1.5, 3, 6, 12, and 16 ppb for the gelatine samples.

### 3.9. Solution Nebulisation ICP-MS

Trace element concentrations of the samples were measured using solution-based ICP-MS [Agilent 8900 Triple Quad (QQQ)] with a helium gas collision cell (Agilent Technologies, Tokyo, Japan) at University of Adelaide. An internal standard, yttrium, was mixed online with the samples to compensate for matrix effects. Blanks were interspersed throughout the analysis session, as well as the measurement of a 50-ppb calibration solution to check the instrument stability. The torch depth was 10 mm, octupole RF power was 170 V and argon carrier gas flow was 1.05 L/min. The measurement was performed using an octupole collision cell, with helium gas flow 5.0 mL/min to avoid polyatomic interferences. Five replicates were acquired for each sample. The data was processed using Agilent MassHunter Data AnalysisTM.

### 3.10. Cell Culture and MTT Cytotoxicity Assay

Immortalised human keratinocytes cell line (HaCaT) was cultured in DMEM (high-glucose, Sigma-Aldrich) supplemented with 10% FBS (Sigma-Aldrich) and 1% Penicillin-Streptomycin Solution (P/S, Life Technologies, Carlsbad, CA, USA), then incubated at 37 °C under a humidified atmosphere of 5% CO_2_/95% air. After 4 passages, cells were detached with trypsin and seeded in a 96-well plate (Corning Costar, Corning, NY, USA) at a density of 50,000 cells per well. HaCaT cells are a commonly used cell line that offer a robust and reproducible model for assessing keratinocyte cytotoxicity by removing inherent donor variability observed when using primary keratinocytes.

To perform a cytotoxicity test, 1 mL of stock suspension of ^67^ZnO-PEG NPs (1 mg/mL) in DMEM was prepared, following ultrasonication for 30 min, then diluted to concentrations ranging from 10 to 100 μg/mL. In addition, solutions of zinc salts, zinc sulphate heptahydrate (ZnSO_4_.7H_2_O; Sigma-Aldrich, Saint Louis, MO, USA), and zinc acetate dihydrate (Zn(C_2_H_3_O_2_)_2_.2H_2_O; Sigma-Aldrich, Saint Louis, MO, USA) were prepared at concentrations adjusted to the same Zn molar content as that of ZnO NPs. Eight wells were tested for each concentration and 8 wells were kept as control untreated cells. The media was removed 24 h later, cells were washed with PBS and then were exposed to freshly prepared dilutions of ZnO NPs for testing concentrations. After 24 h, cells were washed twice with PBS, and the cell viability was determined by MTT (3-(4,5-Dimethythiazolyl)-2,5-diphenyl-2H-tetrazolium bromide) colourimetric assay. Briefly, 100 μL of MTT reagent (0.5 mg/mL in the phenol red-free cell culture medium; DMEM/F12; #D6434, Sigma-Aldrich, Sydney, NSW, Australia) was added to each well, followed by incubation at 37 °C for 1 h to allow precipitation of insoluble formazan crystals. Then the supernatant was carefully discarded and 100 μL of DMSO (dimethyl sulfoxide) was added to each well and left for 10 min in dark on a plate shaker at room temperature to allow violet formazan crystals to be dissolved. The absorbance of the MTT product was measured at 550 nm using a PHERAstar microplate reader (BMG Labtech, Ortenberg, Germany) and the cell viability for each concentration was calculated relative to the untreated cells using the following equation [73]:

(1) $$\mathrm{Viability}\;\left(\%\right)=\frac{\langle absorbance\;of\;treated\;cells\rangle -\langle blank\rangle }{\langle control\rangle -\langle blank\rangle }\;\times \;100\%,$$

〈 〉 denotes mean value.

### 3.11. Determination of Labile Zinc Species in Cell-Mediated Culture Media

HaCaT cells were cultured as described in Section 3.10 and dosed with blank, low (5 μg/mL), mid (25 μg/mL) and high (50 μg/mL) concentrations of ^67^ZnO NPs for 24 h. 0.5-mL aliquots of the cell-conditioned media were placed into thick wall polycarbonate tubes and processed by ultra-centrifugation (Beckman-Coulter, Sydney, NSW, Australia) at 100,000× *g* for 45 min at 4 °C. The supernatants were collected and an aliquot of 180 μL of each sample was added to a black 96-well plate. 20 μL of 2-μM ZP1 solution was added to each well, including the blank, and agitated. Fluorescence was read out from each plate, excitation/emission, 490 nm/530 nm, respectively. The concentration of labile zinc, $C_{Zn}$ in each sample was evaluated by using the following equation:

(2) $$C_{Zn}=k_{d}\frac{F\;-{\;F}_{min}}{F_{max}\;-\;F},$$

where $k_{d}$ stands for the dissociation constant reported being 0.7 nM for ZP1 [74]. F is the fluorescence intensity of the samples after addition of 2-μM ZP1. ${\;F}_{min}$—the fluorescence intensity of the blank DMEM cell culture media containing 50 μM of TPEN (N,N,N,N-Tetrakis(2-pyridylmethyl)-ethylenediamine), *F_max_*—the fluorescence intensity of the blank DMEM cell culture media with 5 mM of added ZnSO_4_.

Alternatively, the ^67^Zn concentration in the sample supernatants was quantified by solution-based ICP-MS, as previously outlined in Section 3.9.

### 3.12. Statistical Analysis

Statistical analysis of obtained data was performed using GraphPad Prism software (version 9.2.0). One-way ANOVA and Mann–Whitney tests were performed on normally and non-normally distributed data, respectively, to determine significance of the mean difference between the blank and ZnO-treated samples. *p*-values < 0.05 were deemed statistically significant.

## 4. Conclusions

Whilst there has been concern about the general safety of zinc oxide nanoparticle sunscreens in topical products, work to date has focussed on whether nanoparticles can be found in the viable epidermis after permeation through the SC and via furrows and the follicles, including by lateral diffusion [75]. These zinc oxide nanoparticles, in turn, release zinc ions when the skin pH is either neutral or acidic and high zinc ion concentrations have been shown to be toxic in isolated keratinocytes [41]. Here, the use of ^67^Zn tagged nanoparticles enabled analysis of exogenous zinc ion concentrations in viable skin after topical application of zinc oxide nanoparticles.

The percutaneously absorbed ^67^Zn was quantified by inductively coupled plasma–mass spectrometry (ICP-MS) and laser-ablation ICP-MS to its elevated concentration in the VE as 1.0 ± 0.3 μg/mL (2.1 ± 0.5 μg per g of dry skin), much lower than our estimated endogenous total zinc ion concentration in the viable epidermis of 4.3 ± 0.7 μg/mL (13 ± 2 μg/g of dry skin). Both are, in turn, much lower than the potentially cytotoxic labile ^67^Zn concentrations of 21–31 μg/mL in serum-supplemented culture media causing keratinocyte HaCaT cytotoxicity. Therefore, the zinc concentration detected in VE with and without ZnO NP application was found to be significantly lower than the HaCaT cytotoxicity threshold. As such, our study validates the estimates recently made by Yamada et al. [18]. Furthermore, our work supports the recent FDA proposal for sunscreen marketing that only two of the 16 currently marketed sunscreens, zinc oxide and titanium dioxide, can generally be regarded as safe and effective [76]. This paper provides strong evidence that the NP form of ZnO sunscreens is safe after topical application to intact human skin.

## Figures and Tables

**Table 1:** Summary of the measured and calculated zinc values in human skin.

	CZ 67n, μg/g	$C_{exZn},$ μg/g	$C_{enZn},$ μg/g	$C_{exZn},$ μg/mL	$C_{enZn},$ μg/mL	IC_50_ *, μg Zn/mL
SC	235 ± 49	360 ± 70	39 ± 5	120 ± 23	13 ± 2	-
VE	2.0 ± 0.5	3.1 ± 0.8	13 ± 2	1.0 ± 0.3	4.3 ± 0.7	-
Dermis	0.9 ± 0.3	1.4 ± 0.5	16 ± 7	0.5 ± 0.2	5 ± 2	-
HaCaT cells	-	-	-	-	-	26 ± 5

* cytotoxicity of ^67^ZnO-PEG NPs, equivalent to 25 μg/mL of ^67^ZnO NPs.

## Data Availability

The data presented in this study are available from the corresponding author on reasonable request.

## Supplementary Materials

The following are available online at https://www.mdpi.com/article/10.3390/ijms222212372/s1.
