# Supplementary material for: Human Epidermal Zinc Concentrations after Topical Application of ZnO Nanoparticles in Sunscreens
Source: Int J Mol Sci. 2021 Nov 16;22(22):12372. doi: 10.3390/ijms222212372 (PMC8618668; doi:10.3390/ijms222212372)
Supplement: Supplementary file 1 [file ijms-22-12372-s001.zip › ijms-1454877-supplementary.pdf]

Supplementary Material

Human epidermal zinc concentrations after topical application of ZnO nanoparticles in sunscreens

Zahra Khabir[a,b], Amy M. Holmes[c*], Yi-Jen Lai[a], Liuen Liang[a,b], Anand Deva[a], Michael A. Polikarpov[d], Michael S. Roberts[c,e§], Andrei V. Zvyagin[a,f§]*

[a] Macquarie University, Sydney, Australia

[b] ARC Centre of Excellence for Nanoscale BioPhotonics,

[c] University of South Australia, Basil Hetzel Institute for Translational Health Research, Adelaide, Australia

[d] National Research Centre "Kurchatov Institute", Moscow, Russia

[e] Diamantina Institute, University of Queensland, Brisbane, Australia

[f] Centre of Biomedical Engineering, Sechenov University, Russia

[*]These authors contributed equally to this work

[§]Correspondence: A/prof Andrei Zvyagin (andrei.zvyagin@mq.edu.au), Prof. Michael Roberts (m.roberts@uq.edu.au)

1. Synthesis of ZnO-PEG nanoparticles (NPs) via chemical co-precipitation method

The experimental procedure consisted of two steps. First, the zinc acetate was synthesized using ZnO micron-sized powder (99.9% Sigma-Aldrich) and acetic acid (ACS reagent, ≥99.7%, Sigma-Aldrich) as precursors. The molar ratio of acetic acid to Zn ions was 2:1. The required amount of ZnO powder was dissolved in acetic acid solution (3.8M) under vigorous stirring at 50 °C to get a clear solution. By further heating, the solution was completely evaporated, and the white powder of zinc acetate remained. The dried zinc acetate powder is collected and is used as a precursor in the synthesis of ZnO nanoparticles. The flowchart below describes the first part:

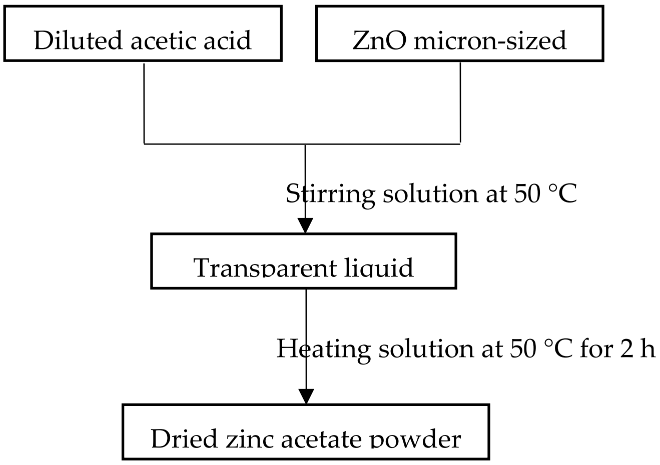

To optimise the synthesis procedure of ZnO NPs in order to tailor the particle size, different molar ratios of $[KOH]/[Zn^{2+}]$ and different amount of Poly(ethylene glycol) (PEG; average molecular weight 200 Da, Sigma-Aldrich) were tried. The general synthesis procedure is shown in the following flowchart and table S1 summarizes the synthesis parameters and characteristics of the synthesized samples (S1-S5). For characterization of samples, transmission electron microscope (TEM, PhilipsCM10, Netherlands), X-ray diffraction (XRD), and Photoluminescence (PL) spectroscopy (Fluorolog-Tau3 spectrofluorometer,

JobinYvon-Horiba, Edison, USA) were used. Fig. S1 shows the TEM images of samples with different synthesis parameters. Fig. S2 presents XRD patterns and PL spectra of the same samples.

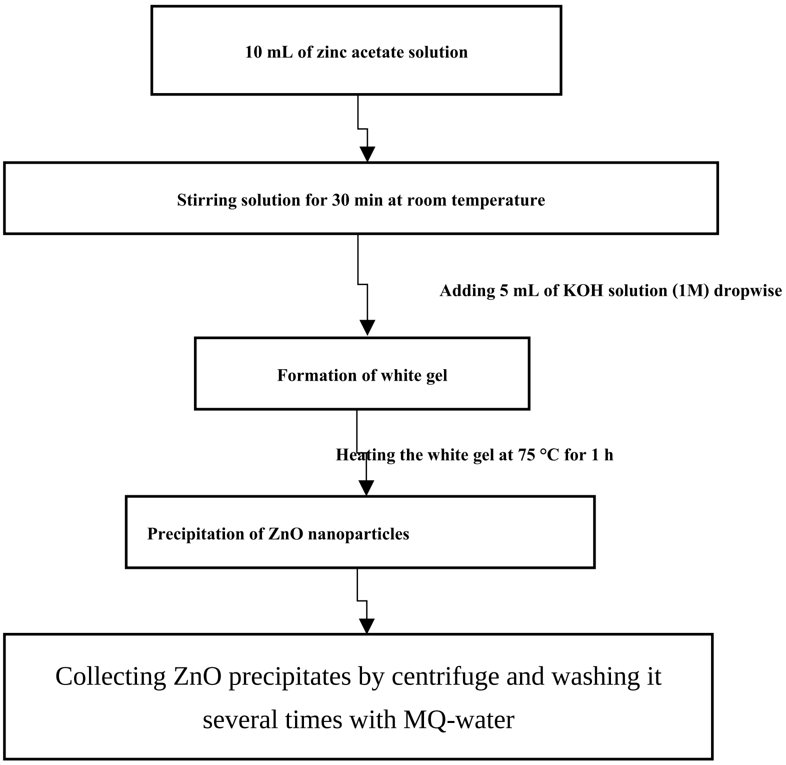

Table S1 Characteristics of the samples and the synthesis parameters.

**Sample ID**	**Crystallite size (nm)**	**Particle size (nm)**	**PEG (μL)**	**[KOH]/[Zn^{2+}]**
S1	18.3	38	222	2
S2	16.59	26	444	2
S3	16.07	25	666	2
S4	15.58	23	444	2.5
S5	-	22	666	3

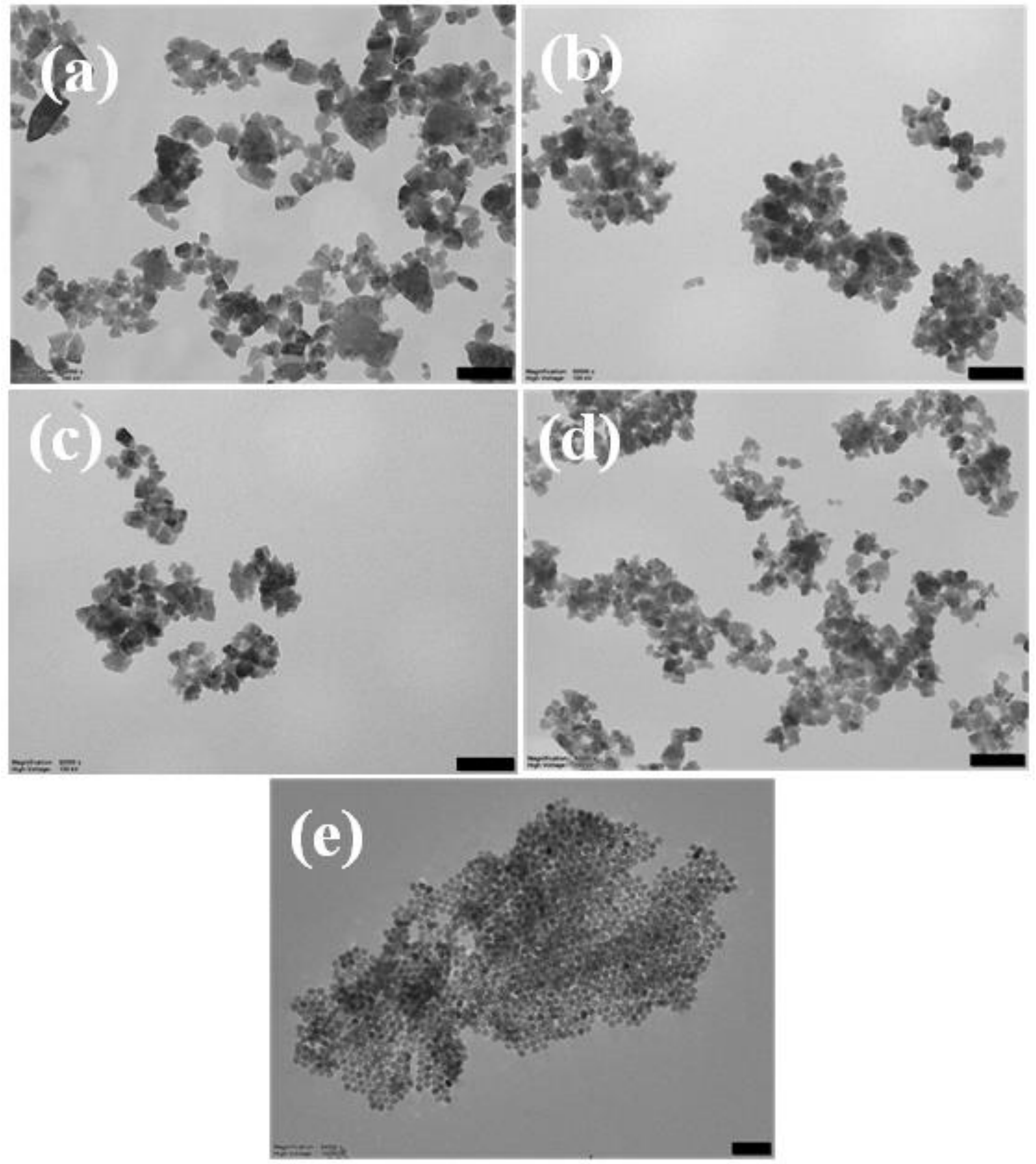

Fig. S1. TEM images of S1 a), S2 b), and S3 c), S4 d), and S5 e). Scale bar 100 nm.

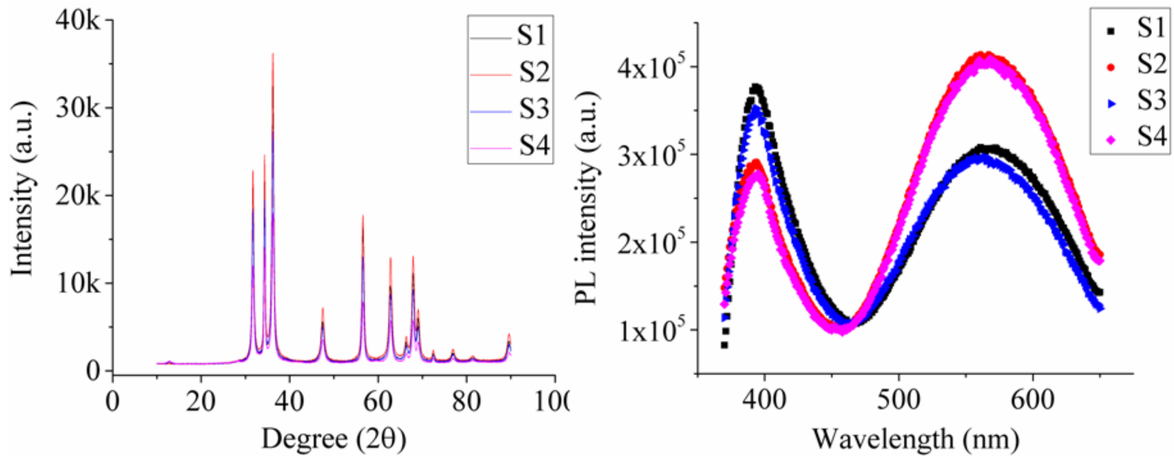

Fig. S2. XRD patterns (left) and PL spectra of ZnO-PEG NPs (right).

2. Determination of normal zinc level in human skin with ICP-MS and LA-ICP-MS

Human skin sample from a female donor (aged 35 years) was cut into small pieces and homogenized in MQ water with a homogenizer (Polytron, USA). After turning the tissue into a paste, it was transferred to the cryomold (Tissue-Tek® Biopsy, square 10×10×5mm) and was dried overnight in a biosafety cabinet. Next day, the sample was collected, divided into two, then prepared for LA-ICP-MS and ICP-MS analyses following the protocols described in sections 2.7 and 2.8, respectively. For ICP-MS analysis, the unknown concentration of zinc was determined through the calibration curve set of 2.5, 5, 10, 25, and 50 ppb.

3. Conversion of μg/g unit to μg/mL for zinc concentration in skin

As explained in the text (subsection 3.4, first paragraph), the water content of skin tissue is 70%. It means, for wet skin tissue, 0.7 g is for water and 0.3 g for dry tissue. Besides, the density of wet skin is 1 g/mL that means 1 g of skin is equivalent to 1 mL. 1 g is equivalent to 0.3 mL for dry skin. Therefore, the concentrations of 13 μg/g and 3.1 μg/g are equivalent to 4.3 μg/mL and 1.0 μg/mL respectively.

4. Determination of labile zinc species in cell mediated culture media with ZP1

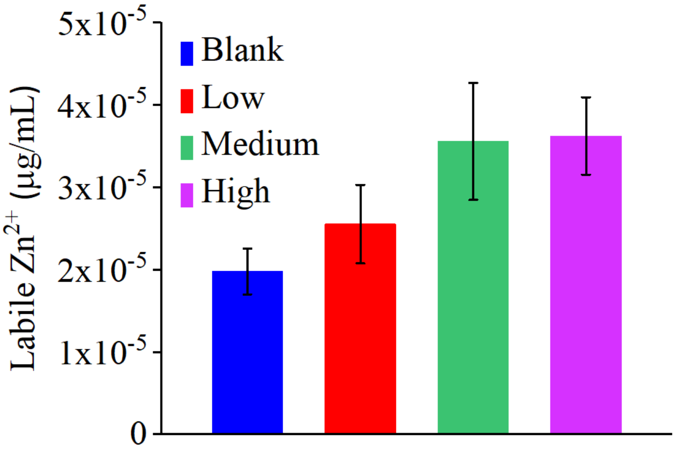

Fig. S3. Labile zinc concentrations measured by ZP1 in HaCaT cell-mediated culture media (24 h) after addition of increasing amounts of ZnO-PEG NP. Blank, Low, Medium and High stand for cell incubation with 0, 5, 25 and 50 μg/mL of ^{67}ZnO-PEG NPs, respectively. The data format: mean ± SD, n = 3.
